# Early rapid weight gain, parental body mass index and the association with an increased waist-to-height ratio at 5 years of age

**DOI:** 10.1371/journal.pone.0273442

**Published:** 2022-09-07

**Authors:** Annelie Lindholm, Gerd Almquist-Tangen, Bernt Alm, Ann Bremander, Jovanna Dahlgren, Josefine Roswall, Carin Staland-Nyman, Stefan Bergman

**Affiliations:** 1 School of Health and Welfare, Halmstad University, Halmstad, Sweden; 2 Research and Development Center Spenshult, Halmstad, Sweden; 3 Department of Pediatrics, Institute of Clinical Sciences, The Sahlgrenska Academy, University of Gothenburg, Gothenburg, Sweden; 4 Child Health Care Unit, Region Halland, Halmstad, Sweden; 5 Department of Regional Health Research, University of Southern Denmark, Odense, Denmark; 6 Department of Pediatrics, Halland Hospital, Halmstad, Sweden; 7 Primary Health Care Unit, Department of Public Health and Community Medicine, Institute of Medicine, The Sahlgrenska Academy, University of Gothenburg, Gothenburg, Sweden; Arizona State University, UNITED STATES

## Abstract

**Background/Objectives:**

Obesity-related adverse health consequences are closely associated with abdominal obesity. Risk factors for overweight and obesity have been studied but there is a lack of information regarding risk factors for abdominal obesity, especially in the preschool population. The aim of the present study was to examine early life risk factors for an increased waist-to-height ratio (WHtR) in children at five years of age and, in addition, to investigate if these risk factors also were associated with overweight or obesity.

**Subjects/Methods:**

The study population comprised 1,540 children from a population-based longitudinal birth cohort study that included 2,666 Swedish children. The children were included if they had complete growth data for the analyses used in this study. Children were classified as having WHtR standard deviation scores (SDS) ≥ 1 or < 1 at five years of age, according to Swedish reference values, and as having body mass index standard deviation scores (BMI_SDS_) for overweight/obesity, or normal weight/underweight according to the International Obesity Task Force criteria. Associations between child-related, socioeconomic status-related, parental health-related and nutrition- and feeding practice-related factors during the first two years and a WHtR_SDS ≥_ 1 or a BMI_SDS_ for overweight/obesity at five years were investigated with logistic regression analyses.

**Results:**

At five years of age, 15% of the children had WHtR_SDS ≥_ 1 and 11% had overweight or obesity. In multivariable analyses, rapid weight gain (RWG) during 0–6 months (OR: 1.90, 95% CI: 1.23–2.95, p = 0.004), maternal pre-pregnancy BMI (1.06, 1.01–1.11, p = 0.019) and paternal BMI (1.11, 1.01–1.21, p = 0.028) were associated with WHtR_SDS ≥_ 1. RWG during 0–6 months (2.53, 1.53–4.20, p<0.001), 6–12 months (2.82, 1.37–5.79, p = 0.005), and maternal pre-pregnancy BMI (1.11, 1.06–1.17, p<0.001) were associated with overweight or obesity.

**Conclusions:**

Early risk factors, including rapid weight gain, are associated with increased WHtR_SDS_ and overweight or obesity at 5 years of age. Preventive interventions should target early RWG and parental overweight and obesity.

## Introduction

Rates of overweight and obesity in children have increased during the last decades, suggesting future global health challenges. In 1990, 32 million children below 5 years of age had overweight or obesity and in 2016 this number had increased to over 41 million [1]. Excess weight in childhood is associated with adverse health consequences later in life, such as metabolic syndrome, diabetes, hypertension and cardiovascular diseases [2]. In both adults and children, obesity-related adverse health consequences are closely associated with abdominal obesity [3, 4], and although these associations need to be further investigated in the preschool population, early signs of metabolic syndrome have been identified as early as during the preschool years [5, 6].

There is no consensus regarding the best measure to identify abdominal obesity in preschool children, but waist-to-height ratio (WHtR) and waist circumference (WC) are two anthropometric measures that have been suggested as good surrogate measures of abdominal obesity and associated cardiometabolic risk in children and adolescents [7–9]. Both methods are easy to perform without any advanced equipment [10, 11].

Although a significant number of studies have examined early life risk factors for overweight and obesity in both childhood and adulthood [12–16], few have investigated risk factors for abdominal obesity, especially in children below six years of age. Given that abdominal obesity has been identified as an important metabolic risk factor, and because these risk factors are increasingly observed in children at younger ages [5, 6], the field needs to be further investigated.

Previous research focusing on abdominal obesity in older children has shown that rapid weight gain (RWG) during the first two years of a child’s life has been associated with both abdominal obesity in adults [17] and overweight or obesity later in life [12, 16, 18]. The reasons for this accelerated weight gain are somewhat unclear, but it is known that breastfed infants have a slower weight-gain trajectory than formula-fed infants, at least in Western settings [19]. One suggested reason for this is differences in milk composition between formula milk and breast milk; for example, protein intake associated with formula milk feeding has been shown to promote RWG [19]. Added sugars in infant formula have also been associated with RWG [20]. In previous research, we have shown that bottle feeding at birth, 3–4 and at six months and night-time meals containing formula milk at 3–4 months were associated with RWG between 0 and six months [21]. Additionally, bottle size, risk of over-feeding, infant-initiated bottle emptying [22] and feeding on schedule [23] have been suggested as possible antecedents of RWG. The relationship between RWG and either gut microbiome or sleep-wake cycles are also being investigated [24].

In addition to RWG, other risk factors for abdominal obesity have been examined; low birth weight has been associated with abdominal obesity, measured by WHtR, in children 3–16 years of age [25], and maternal gestational weight gain has been associated with abdominal obesity in children aged 2–9 [26]. In children aged 8 to 18 years, maternal pre-pregnancy body mass index (BMI) has been associated with abdominal obesity, measured by WC [27]. Female sex is another factor that has been associated with abdominal obesity. Griffiths *et al*. found that 5-year-old UK girls born at the beginning of the millennium had marginally higher waist circumference than boys, and that the girls’ waist circumference had risen more steeply, when compared to children born in the 1970s and 1980s [28].

Regarding risk factors for overweight or obesity other than RWG, higher birth weight [29, 30], maternal pre-pregnancy BMI [12, 29] and maternal gestational weight gain [31, 32] have all been associated with later overweight or obesity in children. Infant nutrition, such as consumption of infant formula [19, 33], milk cereal drink (MCD), a complementary product often served from a bottle and recommended from the age of six months in Sweden, [34] and feeding practices from feeding bottles [22], have all been associated with overweight or obesity later in life. Socioeconomic factors, such as maternal low educational level and smoking have been associated with poorer eating habits [35], and more television watching [36], both of these may lead to excess weight in children. The impact of paternal influences on childhood overweight have been studied, but their effects are still unclear [12].

Given the associations between abdominal obesity and cardiometabolic risk factors in children, and the knowledge gap regarding risk factors for abdominal obesity in the preschool population, the aim of the present study was to examine early risk factors for an increased WHtR at five years of age, in a cohort of Swedish preschool children. A second aim was to investigate if these risk factors also were associated with overweight or obesity at the same age, as measured by BMI.

## Subjects and methods

### Study participants and design

This study is part of the longitudinal and population-based birth cohort study, the Halland Health and Growth Study (H^2^GS). H^2^GS included at baseline 2,666 children– 1,349 males and 1,317 females–born between October 1, 2007 and December 31, 2008 in the county of Halland, in the south-western part of Sweden. During the data collection period, there were 3,860 births in the county. All children were eligible to take part in the study, without any exclusions, and 69% of the families agreed to participate. The families were recruited when they visited child health care centres (CHCCs) for the first time. The coverage for CHCCs in Sweden is approximately 98.4% of children below six years of age. Weight, height and WC were measured on nine occasions during the first five years, with the first measurement point between 0 and 45 days after birth. In conjunction with each measurement point, the parents filled in questionnaires regarding their child’s nutrition, health and lifestyle, along with background data on the family. The study protocol, recruitment process and the representativeness of the sample have been reported in detail elsewhere [37]. In the current study, 1,540 of the 2,666 children were selected, based on if they had complete growth data for the analyses used in this study. This study was approved by the regional ethical review board in Lund, Sweden (Study no.: 299/2007). Written informed consent was obtained from the parents of all the participating children.

**Fig 1:**
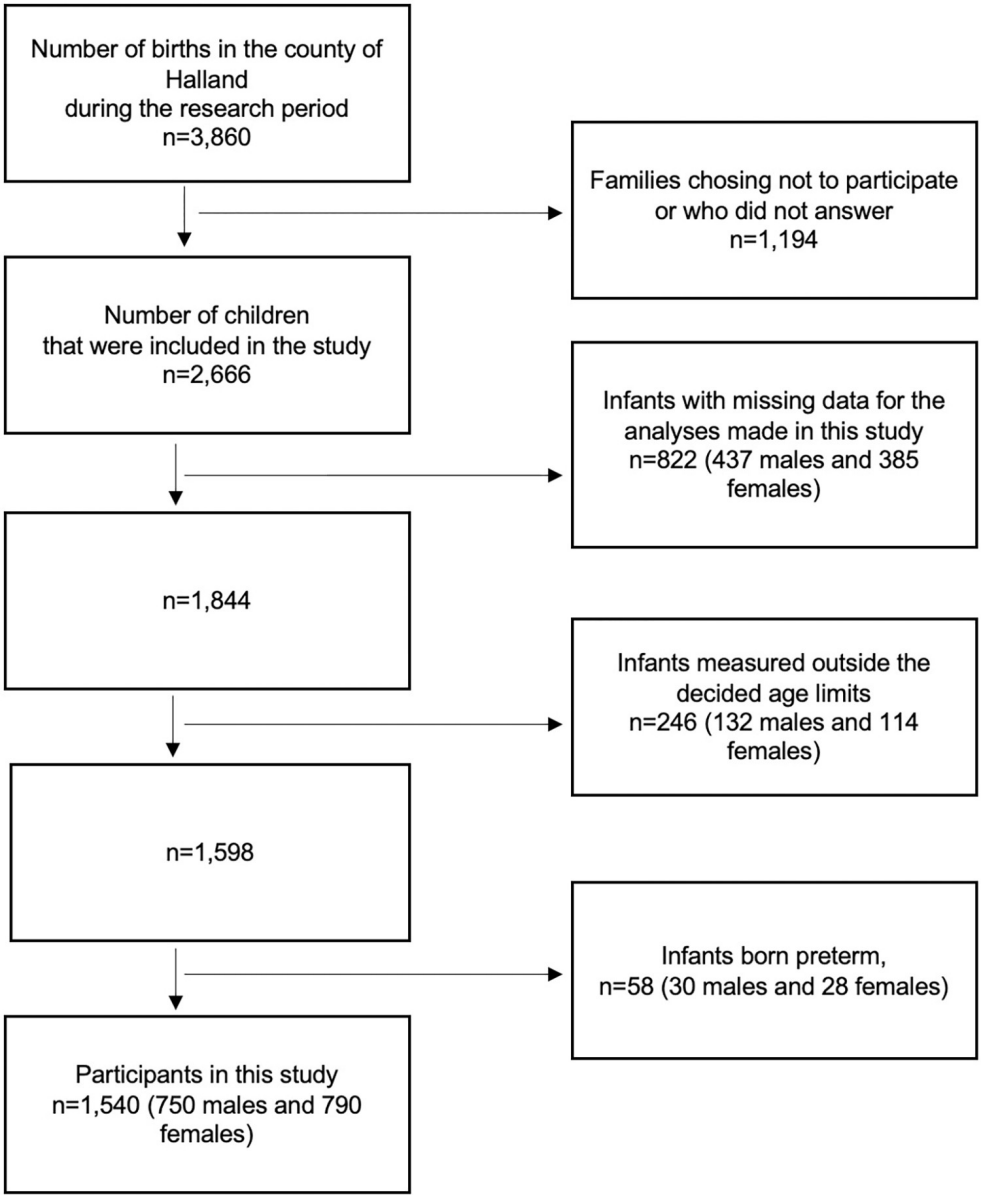
Flowchart of the participants in the study.

Only children with measurements of weight, length and WC at five years of age and information regarding maternal gestational age were included in this study, and those without that information (437 males and 385 females) were excluded. We also excluded children measured outside our set age limit of ± 1.5 months below 12 months of age (except at 0−1 month, where 0–45 days was used instead) and ± 2.5 months thereafter (132 males and 114 females). Additionally, children born preterm (30 males and 28 females) were excluded. The total remaining study population consisted of 750 males and 790 females (Fig 1). A slightly higher proportion of the excluded children (1,126) were male, 53% versus 49%, and had a slightly lower birth weight, 3,495 g versus 3,580 g.

### Auxology and classifications

For gestational age at birth, the date of the mother’s last menstrual period, confirmed by antenatal ultrasound reports, was used. Information regarding birth weight, birth length, sex and gestational age was collected from the Swedish National Medical Birth Register. Birth length was reported to the nearest half centimeter and birth weight was reported to the nearest gram. Anthropometric measurements were performed during the first 45 days after birth, and then at 3–4, 6, 12, 18, 24, 36, 48 and 60 months, by trained child health care nurses at the CHCCs. Infants below 15 kg were weighed without clothes, in a supine position on baby scales. Children above 15 kg were weighed on mechanical or electronic step scales in a standing position without shoes and wearing underwear. Stadiometers were used to measure height. In children below two years of age, height was measured in a supine position and, after two years of age, height was measured in a standing position without shoes. WC was measured without clothes and midway between the lowest rib and the top of the iliac crest, at the end of a gentle expiration. For children below two years of age, WC was measured in a supine position and in a standing position for children two years of age and above. Crude WHtR values were calculated as WC (cm)/height (cm) and were then transformed to sex- and age-specific standard deviation scores (SDS) using the estimated mean and SD functions, based on Swedish reference data [38]. Within the framework of this project, the intra- and inter-operator reliability for WC were studied in children predominantly younger than 24 months. The intraclass correlation coefficients were 0.98, both within and between those who made the measurements, although one of the persons had no experience, apart from a short introduction. Classification of children as having a WHtR_SDS_ ≥1 or <1 was made at the age of five. The children were classified as: small for gestational age (SGA), defined as birth weight or birth length ≤ −2 SDS; appropriate for gestational age (AGA); or large for gestational age (LGA), defined as birth weight or birth length for gestational age ≥2 SDS based on Swedish reference values [39]. RWG was defined as a change > +0.67 in weight SDS, as described by Ong *et al*. [18, 40]. In our study, this change was examined during 0–6 or 6–12 months. This increase in SDS represents the distance between two adjacent centile lines in standard weight growth charts.

Crude BMI values were calculated as weight (kg)/height^2^ (m^2^) and were thereafter transformed to sex- and age-specific SDS, using the estimated mean and standard deviation (SD) functions based on Swedish reference data [41]. The classifications of children as either having overweight/obesity or normal weight/underweight were made at the age of five years and were based on crude BMI cut-off values by the International Obesity Task Force (IOTF) [42]. Crude BMI values were transformed to corresponding BMI_SDS_: 1.26 and 1.20 for overweight and 2.44 and 2.22 for obesity, in males and females, respectively. Corresponding SDS for underweight, the second grade of thinness (the equivalent of BMI <17 at 18 years), were –2.44 and –2.22 for males and females, respectively. Conversion of crude BMI and WHtR values to SDS values was made in MATLAB (v.9.0.0.341360R2016a; The MathWorks, Natick, Massachusetts, USA).

### Risk factors for an increased WHtR at five years of age

The selection of risk factors was based on previous research regarding risk factors for abdominal obesity or overweight/obesity, and available data in this infant cohort [12, 16, 18, 25–28, 43]. The investigated child-related risk factors were sex, birth weight and RWG between 0–6 and 6–12 months. Birth weight and sex were collected from the Swedish National Medical Birth Register, and RWG was calculated based on measurements from the CHCCs. The investigated nutrition- and feeding practice-related risk factors were breastfeeding and bottle feeding at 0–1, 3–4, 6, 12, 18 and 24 months, together with MCD consumption at 6, 12,18 and 24 months. Breastfeeding was reported at every measurement point and divided into predominant, partial or no breastfeeding, based on answers from the questionnaire responses. At each time point, the parents were asked if the child was breastfed or not. The parents who answered yes were asked how many times per day, with three response alternatives: 1–5 times/day, 6–10 times/day or more than 10 times/day. Infants who were breastfed 6–10 times/day or more were considered predominantly breastfed, while the ones breastfed 1–5 times/day were considered partly breastfed. Bottle feeding was reported at every measurement point, and the parents responded as to whether their infant was fed by a bottle or not. The use of MCD was reported from six months of age. Investigated socioeconomic status-related risk factors were parental educational level (with upper secondary school as reference) and parental smoking, both of which were based on answers from the questionnaire responses. Parental health-related risk factors were pre-pregnancy BMI, maternal gestational weight gain, and paternal BMI, all of them were collected from answers from the questionnaire responses.

### Statistics

Unadjusted logistic regression analyses were performed to examine associations between each of the risk factors and WHtR_SDS_ ≥1 or overweight/obesity at five years of age. Variables that showed associations that were considered significant at the < 0.05 level in the univariable logistic regressions were used in the final multivariable models. The dependent variables were children with a WHtR_SDS_ ≥1 versus children with a WHtR_SDS_ <1, or children with overweight/obesity versus children with normal weight/underweight. Variables for maternal pre-pregnancy BMI, maternal gestational weight gain and paternal BMI were used as continuous variables in the analyses. IBM SPSS statistics (v.25.0; IBM Corp, Armonk, New York, USA) was used in all statistical analyses.

## Results

### Study population

When the 1,540 children were classified according to WHtR_SDS_ ≥1 or <1, 15% had WHtR_SDS_ ≥1 and 85% had WHtR_SDS_ <1 at five years of age (Table 1). When they were classified according to BMI_SDS_ for overweight/obesity or normal weight/underweight, 2% (n = 34) had underweight, 87% (n = 1334) had normal weight, 9% (n = 141) had overweight and 2% (n = 31) had obesity, according to the IOTF BMI cut-off values. Regarding birth size, 3% were born SGA, 93% AGA and 4% LGA.

**Table 1:** Characteristics of the study population classified by WHtR_SDS_ and BMI_SDS_.

	WHtR_SDS_		BMI_SDS_	
	≥1 SD	<1 SD	Overweight/obesity	Normal weight
	(n = 231)	(n = 1309)	(175)	(1365)
**At birth**				
MBW ± SD (g)	3605 ± 458	3575 ± 482 p = 0.539	3785 ± 420	3553 ± 479 p<0,001
Missing (n)	11	62	7	66
**Size for gestational age** ^a^				
SGA (n)	8	40	4	44
AGA (n)	214	1211	160	1265
LGA (n)	7	57	10	54
Missing (n)	2	1	1	2
**Gestational age**				
37^0^−37^6^ (n)	13	56	8	61
38^0^−40^6^ (n)	168	958	127	999
41^0^−43^5^ (n)	50	295	40	305
Missing (n)	0	0	0	0
**Maternal age**				
<25 (n)	21	129	16	134
25–35 (n)	156	865	115	906
≥35 (n)	51	303	43	311
Missing (n)	3	12	1	14
**Maternal pre-pregnancy BMI**				
<18.5 (n)	0	43	1	43
18.5–24.9 (n)	142	920	93	969
25–29.9 (n)	53	202	47	208
**≥**30 (n)	25	100	29	96
Missing (n)	11	44	5	49
**Maternal gestational weight gain**				
Less than adequate (n)	38	256	25	269
Adequate (n)	83	443	48	478
Excessive (n)	88	514	86	516
Missing (n)	22	96	16	102
**Paternal BMI**				
<18.5 (n)	5	44	5	44
18.5–24.9 (n)	146	965	121	990
24.9–29.9 (n)	49	196	26	219
**≥**30 (n)	1	3	0	4
Missing (n)	30	101	23	108
**At 5 years, mean ± SD**				
WHtR	0.53 ± 0.0	0.47 ± 0.0 p<0,001	0.52 ± 0.0	0.48 ± 0.0 p<0,001
WHtR_SDS_	1.57 ± 0.5	-0.32 ± 0.8 p<0,001	1.23 ± 0.9	-0.20 ± 0.9 p<0,001
BMI (kg/m^2^)	17.36 ± 1.6	15.35 ± 1.1 p<0,001	18.37 ± 1.2	
BMI_SDS_	1.16 ± 1.0	-0.31 ± 1.0 p<0,001	1.82 ± 0.6	15.30 ± 1.0 p<0,001
				-0.33 ± 0.9 p< 0,001

^a^Classification of size for gestational age was based on values of birth weight and/or birth length. For mean values in birth weight, WHtR, WHtR_SDS_, BMI and BMI_SDS_, the group with WHtR_SDS_ ≥1 was compared with the group with <1 and the group with overweight/obesity according to BMI_SDS_ was compared with the group with normal weight/underweight. Maternal gestational weight gain categories were based on the institute of Medicine (IOM) guidelines by BMI status [44].

MBW, mean birth weight; SD, standard deviation; SGA, small for gestational age; AGA, appropriate for gestational age; LGA, large for gestational age; 37^0^–37^6^, 37 weeks and 0 days– 37 weeks and 6 days; 38^0^–40^6^, 38 weeks and 0 days– 40 weeks and 6 days; 41^0^–43^5^, 41 weeks and 0 days– 43 weeks and 5 days; WHtR_SDS_, waist-to-height ratio standard deviation scores; BMI_SDS_, body mass index standard deviation scores.

### Associations between risk factors and increased WHtR_SDS_ at five years of age

On examining associations between the risk factors and a WHtR_**SDS**_ ≥1 at five years of age with univariable logistic regressions, odds ratios (OR) and 95% confidence intervals (CT) showed that, among the child-related risk factors, RWG during 0–6 and 6–12 months was significantly associated with a WHtR_**SDS**_ ≥1 (Table 2). No associations were found for socioeconomic status-related risk factors. Parental health-related risk factors that showed significant positive associations with an increased WHtR_**SDS**_ were maternal pre-pregnancy BMI and paternal BMI. The nutrition- and feeding practice-related risk factors MCD consumption at 24 months and bottle feeding at 12 and 24 months were significantly positively associated with an increased WHtR_**SDS**_ at five years of age.

**Table 2:** Odds ratios and 95% confidence intervals for risk factors for an elevated WHtR_SDS_ at five years of age.

			Univariable logistic regressions ^a^		
Risk factors	WHtR≥1 SDS	WHtR <1 SDS	OR	95% CI	p
**Child-related risk factors**					
*Gender*					
Male	99	651	1	Ref	
Female	132	658	1.32	1.00–1.75	0.054
*Birth weight in kilograms*	n/a	n/a	1.14	0.85–1.54	0.392
*Rapid weight gain 0–6 months*					
No	74	588	1	Ref	
Yes	105	473	1.76	1.28–2.43	**0.001**
*Rapid weight gain 6–12 months*					
No	157	983	1	Ref	
Yes	22	78	1.77	1.07–2.92	**0.026**
**Nutrition-and feeding practice-related risk factors**					
*Milk cereal drink consumption*					
24 months					
No	60	281	1	Ref	
Yes	105	718	1.46	1.03–2.06	**0.032**
*Bottle feeding*					
*12 months*					
No	27	103	1	Ref	
Yes	165	1007	1.60	1.02–2.52	**0.043**
*Bottle feeding 24 months*					
No	60	268	1	Ref	
Yes	110	748	1.52	1.08–2.15	**0.017**
**Socioeconomic status-related risk factors**					
*Maternal educational level*					
Upper secondary school	100	520	1	Ref	
Elementary school	67	16	1.24	0.69–2.23	0.469
University	102	650	0.82	0.61–1.10	0.182
Other	10	60	0.87	0.43–1.75	0.690
*Paternal educational level*					
Upper secondary school	118	693	1	Ref	
Elementary school	13	69	1.10	0.59–2.07	0.751
University	68	419	0.95	0.69–1.32	0.770
Other	11	55	1.18	0.60–2.31	0.641
*Maternal smoking*					
No	214	1226	1	Ref	
Yes	14	69	1.16	0.64–2.10	0.619
*Paternal smoking*					
No	118	1116	1	Ref	
Yes	25	113	0.20	0.85–2.14	0.202
**Parental health-related risk factors**					
*Maternal pre-pregnancy BMI*	n/a	n/a	1.07	1.03–1.10	**<0.001**
*Maternal gestational weight gain*	n/a	n/a	1.00	0.97–1.03	0.930
*Paternal BMI*	n/a	n/a	1.09	1.02–1.16	**0.007**

^a^The logistic regressions included each of the independent variables separately. Among risk factors related to nutrition- and feeding practices, breastfeeding and bottle feeding at 0–1, 3–4, 6, 12, 18 and 24 months and milk cereal drink consumption at 6, 12, 18 and 24 months were investigated, but significant associations with an elevated WHtR_SDS_ at five years of age were only found for milk cereal drink consumption at 24 months and bottle feeding at 12 and 24 months. A p-value <0.05 was considered significant.

OR, odds ratios; 95% CI, 95% confidence intervals; p, p value; WHtR, waist-to-height ratio; SDS, standard deviation scores; BMI, body mass index.

On examining associations between risk factors and an elevated WHtR_SDS_ at five years of age with multivariable logistic regression, where all variables were controlled for each other, OR and 95% CI showed that RWG between 0–6 months, maternal pre-pregnancy BMI and paternal BMI were significantly positively associated with an increased WHtR_SDS_ at five years of age (Table 3).

**Table 3:** Odds ratios and 95% confidence intervals for risk factors for an elevated WHtR_SDS_ in children at five years of age.

Risk factors	Multivariable logistic regressions ^a^		
	OR	(95% CI)	P
**WHtR**_**SDS**_ ≥**1—WHtR**_**SDS**_ **<1**^b^			
Rapid weight gain 0-6m	1.90	(1.23–2.95)	**0.004**
Rapid weight gain 6-12m	1.85	(0.93–3.69)	0.081
Maternal pre-pregnancy BMI	1.06	(1.01–1.11)	**0.019**
Paternal BMI	1.11	(1.01–1.21)	**0.028**
Milk cereal drink 24m	1.17	(0.52–2.62)	0.713
Bottle feeding 12m	1.17	(0.57–2.42)	0.668
Bottle feeding 24m	1.54	(0.67–3.55)	0.308

^a^In the logistic regressions, the variables were all controlled for each other. No other variables were included in the models, besides those presented in the table.

^b^The children were classified as having waist-to-height ratio standard deviation scores (WHtR_SDS_) _≥_1 or <1 according to Swedish reference values (38). OR, odds ratios; 95% CI, 95% confidence interval; p, p value; m, months; BMI, body mass index. A p-value <0.05 was considered significant.

### Associations between risk factors and overweight or obesity at five years of age

On examining the variables that were positively associated with a WHtR_SDS_ ≥1 at five years of age, regarding their association with overweight or obesity at age five with univariable logistic regression analyses, OR and 95% CI showed that three of them, RWG between 0–6 months, RWG between 6–12 months and maternal pre-pregnancy BMI were significantly positively associated with overweight or obesity (Table 4).

**Table 4:** Odds ratios and 95% confidence intervals for risk factors for overweight or obesity in children at five years of age.

Risk factors	Univariable logistic regressions ^a^			Multivariable logistic regressions ^b^		
	OR	(95% CI)	p	OR	(95% CI)	p
**Ow/Ob- Nw/Uw** ^c^						
Rapid weight gain 0-6m	1.95	(1.36–2.81)	**<0.001**	2.53	(1.53–4.20)	**<0.001**
Rapid weight gain 6-12m	2.03	(1.19–3.47)	**0.009**	2.82	(1.37–5.79)	**0.005**
Maternal pre-pregnancy BMI	1.12	(1.09–1.16)	**<0.001**	1.11	(1.06–1.17)	**<0.001**
Milk cereal drink 24m	1.17	(0.78–1.75)	0.454	2.23	(0.96–5.21)	0.064
Bottle feeding 12m	1.26	(0.73–2.17)	0.404	1.91	(0.85–4.26)	0.116
Bottle feeding 24m	0.87	(0.57–1.33)	0.512	0.48	(0.19–1.22)	0.124

^a^The univariable logistic regressions included each of the independent variables separately, and in the

^b^multivariable logistic regressions they were all controlled for each other. No other variables were included in the models besides those presented in the table.

^c^The children were classified as having overweight or obesity or as having normal weight or underweight according to BMI cut-off values by the International Obesity Task Force [42].

OR, odds ratios; 95% CI, 95% confidence interval; p, p value; Ow/Ob, overweight/obesity; Nw/Uw, normal weight/underweight; BMI, body mass index.

In the multivariable model, the same variables, RWG during 0–6 months, RWG during 6–12 months and maternal pre-pregnancy BMI were significantly positively associated with overweight or obesity at five years of age. None of the nutrition- and feeding practice-related risk factors showed significant associations with overweight or obesity at five years of age.

## Discussion

In this longitudinal birth cohort study, 15% of the children had a WHtR_SDS_ ≥1 at five years of age, and three risk factors were significantly positively associated with this increased WHtR_SDS_: RWG during 0–6 months, maternal pre-pregnancy BMI and paternal BMI. Regarding the second aim, which was to investigate if these risk factors also were associated with overweight or obesity, as measured by BMI at five years of age, 9% of the children had overweight and 2% had obesity. RWG between 0–6 months, between 6–12 months and maternal pre-pregnancy BMI were significantly positively associated with this excess weight in the children.

RWG during 0–6 months was positively associated with both an increased WHtR_SDS_ and overweight or obesity at five years of age and RWG during 6–12 months was associated with overweight or obesity. In a number of previous studies, RWG during the first two years of life has been associated with childhood overweight or obesity later in life [12, 16, 18]. The number of studies examining associations between RWG and abdominal obesity are fewer, but these associations have been found in two-year-old children [45]. In a recent review by Zheng *et al*., regarding associations between RWG and health risks later in life, the authors hypothesised that abdominal obesity potentially may be the factor linking RWG and later health risks [16]. Our results regarding early RWG as a risk factor for an increased WHtR_SDS_ are concordant with this hypothesis and indicate that the first six months of life is a crucial period that needs further examination regarding these associations. This period of life is also associated with a large accumulation of fat mass in the growing child [46]. Children have predominantly subcutaneous fat during their first months, but Moreno *et al*. showed by sonography that in the second year of life, there was a pronounced increase in pre-peritoneal fat thickness, a proxy for abdominal visceral fat, in copmarison with subcutaneous fat thickness that only increased to a smaller extent [47]. It has also been shown that children with RWG during their first two years had increased pre-peritoneal fat mass [45]. In older children, 6–14.9 years, it has been suggested that increased adiposity is associated with increased fat deposition in the abdominal area [48].

Maternal pre-pregnancy BMI was another risk factor that was positively associated in our current study with an increased WHtR_SDS_ and with overweight or obesity. Associations between maternal pre-pregnancy BMI and overweight or obesity in children have been reported in previous studies [12, 29], but the number of studies examining its effect on abdominal obesity are limited. However, the association has been found in older children, aged 8 to 18 [27]. Another study, with 10-year-old children from the Netherlands, also found associations between maternal pre-pregnancy BMI and abdominal fat [49]. The underlying mechanisms behind these associations are still unclear and have mainly been investigated in small human studies and animal studies [50]. This indicates that more studies regarding the mechanisms behind maternal pre-pregnancy BMI and an increased WHtR_SDS_ in preschool children and in older children are needed.

Regarding paternal BMI and its association with an increased WHtR_SDS_ in children, it has been shown that fathers with obesity or abdominal obesity (WC >102 centimeters) were more likely to have children born SGA [51], and several studies have shown that children born SGA are more prone to RWG, abdominal obesity and insulin resistance [52]. Due to the population-based design of this study, only a few percent of the children included were born SGA, and therefore it was not possible to examine if a similar association existed in the current study. There are also conflicting results. A study in six-year-old children from the Netherlands showed no association between paternal BMI and subcutaneous or pre-peritoneal abdominal fat [53].

There were significantly positive associations between MCD consumption at 24 months, as well as bottle feeding at 12 and 24 months, and an increased WHtR_SDS_ in the univariable analyses; however, these associations were not found in the multivariable analyses. Regarding overweight or obesity, there were no associations with the nutrition- and feeding practice-related risk factors. In other studies, both bottle feeding [12] and the consumption of MCD have been associated with overweight or obesity in children [34], and bottle feeding has been associated with RWG during the first year [54]; however, our results indicate that early RWG and parental overweight are more important risk factors for increased WHtR_SDS_ and overweight or obesity at five years than are nutrition and feeding-practice-related factors. However, associations between bottle feeding and formula feeding-practices with RWG [22, 54], suggests that such factors may have an indirect effect. Furthermore, follow-up studies in this population may reveal whether early nutrition and feeding practices are associated with abdominal obesity, overweight or obesity later in life.

A limitation of the current study was that we had to exclude children because of missing growth data at five years of age, or because they were measured outside our set age limit. The main reason for this exclusion was because we wanted to include only measurements close to the measurement points, and wider limits for this would have affected precision. We made multiple independent assessments of associations; this represents a risk factor for overstating associations, and could be considered a limitation. The p-values in our results must therefore be considered with caution. An additional limitation was that our study was based on Swedish children, and this must be considered when generalising the results to populations with another ethnic composition and different impact of socioeconomic factors. The main strengths of this study were the population-based longitudinal design, following a large birth cohort over time, and that all measurements were made by trained child health care nurses.

## Conclusion

We found that rapid weight gain during 0–6 months and parental BMI were positively associated with a WHtR_SDS_ ≥ 1 at five years of age. Rapid weight gain during both 0–6 months and 6–12 months and maternal pre-pregnancy BMI were associated with overweight or obesity at the same age. Preventive interventions regarding abdominal obesity and childhood excess weight should target early rapid weight gain, parental overweight and parental obesity. Further research is required to uncover whether these risk factors are associated with subsequent abdominal obesity, overweight or obesity in older children and adolescents.
